# Editorial

**Published:** 1860-09

**Authors:** 


### EDITORIAL.

### ILLINOIS STATE MEDICAL SOCIETY.

In accordance with the instructions of the State Medical Society at its last annual meeting, *notice* is hereby given that the annual meeting for 1861, will be held on the *First Tuesday* in *May* next, in the City of Jacksonville.

N. S. DAVIS, M. D.,

*Per. Sec'y Illinois State Medical Society.*

Chicago, Aug. 28th, 1860.

### MEDICAL SCHOOLS IN CHICAGO.

The Annual Announcements of the two Medical Schools in this city have been issued, and the time is fast approaching for the Commencement of their respective Annual College terms.

The Announcement of the *Rush Medical College*, indicates no change since the last term, either in the Faculty or the general course of instruction. To such students as are desirous of skimming over all the branches, or more properly, of following the lecturers over a part of each branch for sixteen weeks, without thoroughly reviewing any, the Rush Medical College affords as strong inducements as any similarly organized school in the country. Its regular term commences on the *first* Monday in November, and continues sixteen weeks; but a preliminary course of Lectures and Clinical instruction will be given during the month of October.

The Announcement of the Medical Department of Lind University, also shows no change in the plan of organization, and the courses of instruction adopted by that institution, and but one change in the Faculty. When the first Annual Announcement of the University was issued, the Chair of Materia Medica was vacant. Subsequently the Chair was filled by transferring other members of the Faculty in such a way as to give the Chair of Anatomy to Prof. T. Deville. At the close of the term, Prof. Deville resigned, and the other members of the Faculty were restored to the Chairs originally assigned to them, leaving that of Materia Medica again vacant. This Chair has now been filled by the appointment of A. L. McArthur, M. D., of Joliet, Ill.

Prof. McArthur pursued the study of medicine in this State, and several years since graduated in Rush Medical College. Subsequently he attended the Schools and Hospitals of Philadelphia during the full college term, and received an additional Diaploma from the University of Pennsylvania. Since that time he has resided in Joliet, where he has acquired an extensive and profitable practice. Though in the prime of life, he will bring to the duties of his Chair, a degree of scholarship and practical experience which cannot fail to give him a high reputation as a teacher. The regular lecture term in the

University commences on the second Monday in October, and continues until the first Monday in March. It is founded on the principle, that college instruction in medicine, like that in all other sciences, should be progressive and adapted to the stage of advancement of the student.

Hence, each term is divided into Junior and Senior departments. The first embraces Descriptive Anatomy, Physiology and Histology, Materia Medica, Pathology and Public Hygiene, and Inorganic Chemistry, and is designed for students attending their first course. The second or Senior department embraces Surgical Anatomy, Organic Chemistry, and Practical Medicine, Surgery, and Obsteterics with diseases of women and children, and full courses of Clinical Medicine and Surgery. Practical Anatomy in the dissecting room, and a full course of lectures on Medical Jurisprudence are open to the students in both departments. By adopting this plan, the Trustees and Faculty of the University, designed to accomplish the following important objects: First, to induce a more systematic or methodical mode of pursuing the study of Medicine. Second, to compel the student to acquire a competent knowledge of those more elementary branches, which must constitute the foundation of all correct medical education, before engrossing the mind with the details of the practical branches. Third, by increasing the number of Professorships, dividing and lengthening the term, to insure full instruction in the very important departments of Organic Chemistry, Histology, Surgical Anatomy, and Medical Jurisprudence, branches that are very briefly discussed or not taught at all in most of the Medical Colleges of this country. Fourth, by requiring the student to attend a smaller number of lectures each day, and increasing the length of time, to insure him sufficient time to digest what he hears, and to acquire a mental discipline of the utmost value to the practising physician. Fifth, to give that prominence to *Clinical* Medicine and Surgery, which their importance entitles them to, by devoting an hour every morning to Clinical instruction in the Mercy Hospital, and an additional hour every Wednesday and Saturday at the Dispensary in the College. That the actual attainment of these objects would greatly elevate the standard of Medical education, and correspondingly benefit both the profession and the community none can deny. That the plan of organization and instruction adopted by the Lind University, will practically accomplish these purposes, the experience of the last session afforded abundant proof. We learn from the Secretary that the prospects are good for a largely increased class the coming lecture term. It should be mentioned that while the Junior and Senior departments are kept perfectly distinct, and first course students are *required* to take the full Junior course and sustain a thorough examination on the branches taught therein, yet, the lecture hours are so arranged in the two departments, that any student in the junior class who wishes to spend more time in the Lecture room, can attend any part on the whole of the courses on Practical Medicine, Surgery, and Obstetrics, without additional charge. And on the other hand, the students in the senior class can also, if they choose, attend again any part on all of the courses on Anatomy, and Physiology and Histology, in the junior department.

We say then, with all candor and sincerity, to the profession of the north-west, that, *Chicago*, with two medical schools, one on the ordinary plan and the other on a plan embracing a more methodical and extended system of instruction than any other school in this country; with three Hospitals and two or three Dispensaries accessible for clinical instruction, presents in every respect as good advantages for acquiring a complete and thoroughly practical medical education as any other city in the Union.

---

*Museum and Library of the Medical Department of the Lind University.*—We should have mentioned in giving an account of the Medical Schools in this city, that the Trustees of the University have secured for the museum of that school, the whole collection of Anatomical preparations, etc., brought from Paris by Prof. Deville. Such additions have also been made in other departments, as to render the means of illustration in all the branches of medical science very full and satisfactory. The Library of the same school has also been increased to nearly 1,000 volumes, accessible to the medical classes.

